# GNSS/LiDAR-Based Navigation of an Aerial Robot in Sparse Forests

**DOI:** 10.3390/s19194061

**Published:** 2019-09-20

**Authors:** Antonio C. B. Chiella, Henrique N. Machado, Bruno O. S. Teixeira, Guilherme A. S. Pereira

**Affiliations:** 1 Graduate Program in Electrical Engineering, Universidade Federal de Minas Gerais (UFMG), Belo Horizonte-MG 31270-901, Brazil; acbchiella@ufmg.br (A.C.B.C.); henriquenm@ufmg.br (H.N.M.); brunoot@ufmg.br (B.O.S.T.); 2 Department of Mechanical and Aerospace Engineering, West Virginia University, Morgantown, WV 26506, USA; guilherme.pereira@mail.wvu.edu

**Keywords:** forest flight, surveillance, robust state estimation, sensor fusion, motion planning

## Abstract

Autonomous navigation of unmanned vehicles in forests is a challenging task. In such environments, due to the canopies of the trees, information from Global Navigation Satellite Systems (GNSS) can be degraded or even unavailable. Also, because of the large number of obstacles, a previous detailed map of the environment is not practical. In this paper, we solve the complete navigation problem of an aerial robot in a sparse forest, where there is enough space for the flight and the GNSS signals can be sporadically detected. For localization, we propose a state estimator that merges information from GNSS, Attitude and Heading Reference Systems (AHRS), and odometry based on Light Detection and Ranging (LiDAR) sensors. In our LiDAR-based odometry solution, the trunks of the trees are used in a feature-based scan matching algorithm to estimate the relative movement of the vehicle. Our method employs a robust adaptive fusion algorithm based on the unscented Kalman filter. For motion control, we adopt a strategy that integrates a vector field, used to impose the main direction of the movement for the robot, with an optimal probabilistic planner, which is responsible for obstacle avoidance. Experiments with a quadrotor equipped with a planar LiDAR in an actual forest environment is used to illustrate the effectiveness of our approach.

## 1. Introduction

**Figure 1:**
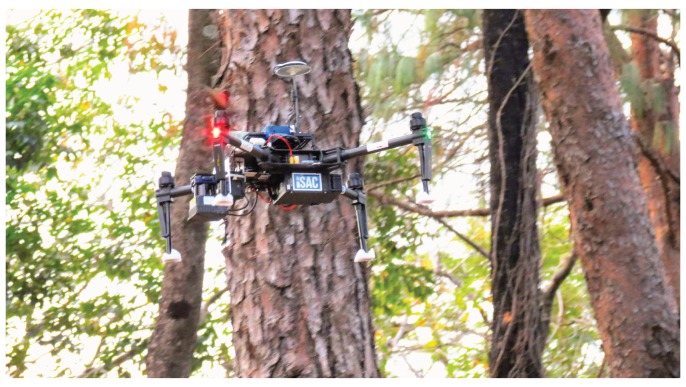
Aerial vehicle used in this work flying in a sparse forest.

Robot navigation in forests is a big challenge, mainly due to the several obstacles existent in such environments, such as tree trunks, bushes and uneven or swamped terrains. Ground robots that can navigate in forests are usually expensive, due to their complex and adaptive locomotion systems [1,2]. In this scenario, autonomous Micro Air Vehicles (MAVs), such as electric drones, appear as a viable and cost-effective alternative, as they are able to fly below the canopies of the trees and execute several missions such as surveillance [3], search and rescue [4], ecological monitoring [5], and forest management [6]. Although the forest terrain has low or no influence in the motion of the MAVs, forest environments may be GNSS (Global Navigation Satellite System)-denied environments, preventing the use of standard and commercial localization systems. Also, in forests, the presence of several unknown obstacles imposes the need for efficient online obstacle avoidance systems to keep the flight safe and allow the completion of the robotic task. This paper presents solutions for both localization and motion control of a drone inside a sparse forest. In this context, we consider a forest to be sparse if the average distance between trees is large enough to (i) allow a safe flight among the trees and (ii) permit GNSS signals to be sporadically available. A picture of our MAV flying in a sparse forest using our integrated solution is shown in Figure 1.

Several recent works have addressed the problem of flying in a forest. Cui et al. [7], for example, developed an autonomous navigation system for a quadrotor drone in forests using Simultaneous Localization and Mapping (SLAM) to construct a map and localize the robot. The authors assume that GNSS signals are never available inside the forest. The vehicle is equipped with an Attitude and Heading Reference System (AHRS) and a LiDAR (Light Detection and Ranging) sensor that is used in a LiDAR odometry (LO) algorithm. Although the inertial sensors in the AHRS are used in the forecast step of a Kalman filter (KF), LO-based estimates of the drone velocity and magnetometer-based heading are used in its data assimilation step. After the robot is localized on the map, the system plans and controls the vehicle through a safe trajectory.

Due to their complementary characteristics, the combination of inertial and GNSS measurements is the standard approach for most navigation solutions for drones and other small flying vehicles. However, for long drop-out periods of the GNSS, the lower-grade AHRS, which are normally embedded in these aerial vehicles, are not sufficient to estimate the robot position and velocity. Therefore, it is necessary to combine information from other exteroceptive sensors such as cameras and LiDAR as is done by the authors of [7] and other authors [8,9].

Shen et al. [8] proposed a modular and extensible multisensor fusion algorithm based on the unscented Kalman filter (UKF). The proposed algorithm is able to combine information from a wide variety of sensors. However, it is not proposed as a solution for the time-varying uncertainty of the sensors, which is the case when, for instance, the GNSS signal changes from fully available to unavailable. Chambers et al. [9] also proposed a multisensor fusion algorithm able to combine relative and absolute information. The authors proposed the use of a chi-squared test to reject outliers, common, for example, in LiDAR odometry data. Despite the algorithm being able to reject outliers in the measurements, it is vulnerable to slow drift error in the measurements, as identified by the authors.

The combination of inertial and visual/LiDAR odometry for state estimation is commonly divided into loosely and tightly coupled approaches. The later jointly estimates the vehicle states and the position of the detected visual landmarks [10,11]. On the other hand, loosely coupled approaches compute the vehicle motion by comparing sequential images (scans) [12,13], and then the estimated motion is used in the fusion algorithm. In the present work, a loosely coupled LO is used because of its smaller computational burden. However, two challenges arise in this strategy: the first is how to combine relative information from LO with absolute information from GNSS system into a fusion architecture; the second is how LO and GNSS errors are modeled in the standard Kalman filter-based fusion algorithms, since they are usually corrupted with time-varying noise, such as outliers and slow drift, which are not easily modeled by Gaussian variables.

For the problem of combining relative and absolute measurements, there are typically three approaches in the literature: using LO measurements as pseudo-global information [12], numerical differentiating the relative motion to compute velocity [7], and applying the so-called stochastic cloning approach [14]. In practice, pseudo-global position drifts with time, and the numerically computed velocity is a poor approximation of the actual velocity. Thus, some authors [8,9,15] advise that one should rather consider LiDAR-odometry as a relative measurement and use stochastic cloning. This is basically a state augmentation technique, in which two instances of the same states, at different time instants, are concatenated in the state vector. These two instances are then used to define a measurement model that explicitly considers relative information given by LO. Our strategy uses the stochastic cloning approach within a UKF.

The classical way to deal with time-varying uncertainty of measurements is the use of adaptive filters, in which the statistical parameters that characterize the uncertainty are jointly estimated with the dynamic states of the system. In this context, approaches based on the techniques, namely, covariance matching (CM) [16], Interacting Multiple Models (IMM) [17], and covariance scaling (CS) [18], were investigated. Among these methods, the covariance matching approaches yield improved results in the estimation of the covariance matrix for Gaussian distribution, if compared to the CS approach, and also with greater simplicity compared to approaches based on multiple models [19]. However, in the presence of outliers, its performance can be damaged. In such case, statistical tests, such as chi-squared test, can be used to identify and reduce the influence of outliers [9].

The first contribution of this work is a LiDAR-based odometry for forest environments. Although similar to the work by the authors of [20], we refine the motion estimate with an iterative closed point (ICP) algorithm. The second contribution is the extension of the Quaternion-Based Robust Adaptive Unscented Kalman Filter (QRAUKF), proposed in our previous work only for attitude estimation [21]. In the present paper, this method is used to estimate the complete state of the MAV by combining measurements from relative and absolute sensors, namely, LiDAR-based odometry, GNSS, and AHRS. The proposed sensor fusion algorithm can handle the time-varying uncertainty of measurements, such as outliers and slow varying errors. Note that some preliminary results of these contributions were initially presented in our conference paper [22].

To fulfill the requirements of a complete navigation system, we integrate the proposed state estimation approach with the motion planning strategy proposed by Pereira et al. [23]. In this strategy, a continuous vector field, such as the one proposed by Gonçalves et al. [24], which is meant to encode the drone task and is constructed over the environment. This vector field only indicates the main direction of movement for the vehicle and completely ignores small obstacles, such as trees. These obstacles are considered in a lower level of the strategy, which is based on an optimal motion planner that generates trajectories that both follows the vector field and avoids the obstacles. In this regard, the third contribution of this paper is the development an efficient LiDAR-based object detection and its integration with the optimal motion planner, which in our case is a slightly modified version of RRT*, an asymptotically optimal version of the Rapidly Exploring Random Tree (RRT) planner [25]. In this paper we use a 2D vector field and a 2D version of RRT* to fly the robot on a plane parallel to the ground. The present paper is one of the few works that consider a complete navigation system for autonomous aerial vehicles in forests. Another example is the work by the authors of [7], which, in addition to the localization system discussed before, also presents a strategy that uses the estimated information to close the loop with a motion planner. In this sense, the main difference between the work by the authors of [7] and our work is that we have a target curve, where the work by the authors of [7] has a setpoint in 2D. Although the authors also use a two-level planner, their higher level is performed by an A* algorithm that runs for each laser data obtained. Their lower level is a vector field histogram (VFH) method, which is a reactive approach. Although it is difficult to compare both strategies due to the fact that our goal is to follow a curve instead of reaching a setpoint, we believe that having a deliberative approach, such as RRT*, in the lower level of the planner can yield shorter and smoother global paths.

**Figure 2:**
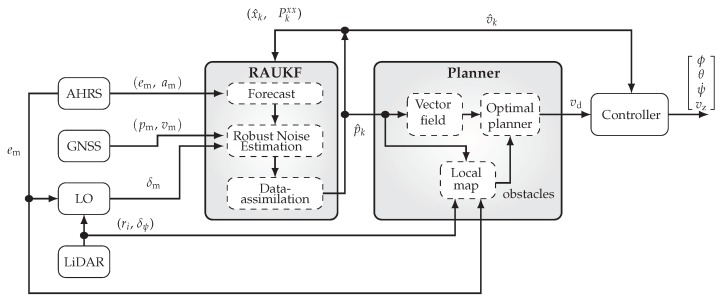
Block diagram of the proposed navigation solution. Data from the available sensors are combined using a robust and adaptive version of UKF (RAUKF). The filter outputs and estimates of position ${\hat{p}}_{k}$ and velocity ${\hat{v}}_{k}$, feed the motion control system, which consists of a path planner and a velocity controller. The controller outputs are the MAV roll ϕ, pitch θ, yaw rate $\dot{\psi }$, and vertical velocity $v_{z}$, with respect to NED frame.

Figure 2 shows the block diagram of the proposed navigation solution. The blocks representing the available measurements of AHRS, GNSS, and LO are discussed in Section 2. In Section 3.1, the laser-based odometer block, LO, is detailed. Section 3.2 presents the mathematical model used in our sensor fusion approach, represented in Figure 2 by the block RAUKF. This approach is detailed in Section 3.3. The motion control system, responsible to plan and drive the robot through the target path is presented in Section 4. Finally, Section 5 presents experimental results executed in a forest environment.

## 2. Problem Statement and Proposed Solution

We address the problem of autonomous navigation of a rotorcraft micro aerial vehicle (MAV) flying in forests. We assume that the forest is sparse, allowing the vehicle to safely fly among the trees and GNSS signals to be detected in some parts of the forest. For a more precise definition, if we assume to have a circular vehicle with one meter diameter, then we consider that a sparse forest has an average distance between two trees of at least four meters.

We also assume that the vehicle is equipped with an AHRS (a GNSS), which may fail when the vehicle is flying inside a forest, and a planar LiDAR whose plane is parallel to the ground. The LiDAR may need to be mounted on a gimbal to make sure its plane is parallel to the ground during the vehicle acceleration or deceleration. From these measurement systems, the following information may be available: (i) AHRS provides the attitude of the vehicle represented as the unit quaternion $e_{m}={\left[e_{0,m}\;e_{1,m}\;e_{2,m}\;e_{3,m}\right]}^{T}\in H_{1}$, with respect to the north-east-down (NED) coordinate frame, and acceleration $a_{m}={\left[a_{x,m}\;a_{y,m}\;a_{z,m}\right]}^{T}$, with respect to the vehicle’s body coordinate frame; (ii) GNSS provides global position $p_{m}={\left[p_{N,m}\;p_{E,m}\;p_{D,m}\right]}^{T}$ and velocity $v_{m}={\left[v_{N,m}\;v_{E,m}\;v_{D,m}\right]}^{T}$ with respect to NED. In this paper we assume that other altitude sensors, such as a barometer, may be also used to compute $p_{D,m}$; and (iii) LiDAR provides distance $r_{i}$ and relative bearing $\delta_{\psi }$ of the environment obstacles. A LiDAR odometry system will use such data to provide estimates of position increments $\delta_{m}={\left[\delta_{N,m}\;\delta_{E,m}\right]}^{T}$ with respect to NED. In our notation "m" denotes on-board measurements. LiDAR measurements will also be used to detect and avoid the obstacles found in the vehicle path.

Figure 2 shows a block diagram of the proposed solution. Basically, the information provided by the sensors is combined by a UKF-based sensor fusion algorithm and the combined information is used by the motion control system, which consists of a path planner and a velocity controller. The path planner computes the vehicle path and uses the information from the LiDAR to construct a local map for collision avoidance. Then, the velocity controller drives the vehicle through the planned path. It is important to mention that, although we assume to have 2D LiDAR-odometry information, the proposed system is able to estimate the states of the vehicle and to perform its guidance and control in 3D. For this paper, however, we use a 2D motion planner that only generates paths at a fixed height parallel to the ground.

Fortunately, most of the drones found in the market today are equipped with AHRS, GNSS, or some combination of both that will deliver part of the data required by our approach. A LiDAR sensor, which is currently becoming lighter and less expensive, must be installed in such vehicles to complete the set-up necessary for our approach.

## 3. Localization

This section presents our solution for MAV localization in forests. We start by presenting the LiDAR based odometry.

### 3.1. LiDAR-Based Motion Estimation in Forests

LiDAR-based odometry is a motion estimation technique that uses the matching between consecutive laser scan data to estimate the incremental motion of the vehicle. In a forest, raw laser measurements do not have much information, as most of the measurement beams do not hit any obstacle. In such a way, more information can be extracted from measurements by detecting environmental features. If this is done efficiently, the detection of features can decrease considerably the amount of data to be processed for motion estimation, thus reducing the computational burden of the entire system. The feature-based laser-odometer algorithm used in this work is mainly composed of two steps: (i) feature extraction and (ii) incremental motion estimation.

**Figure 3:**
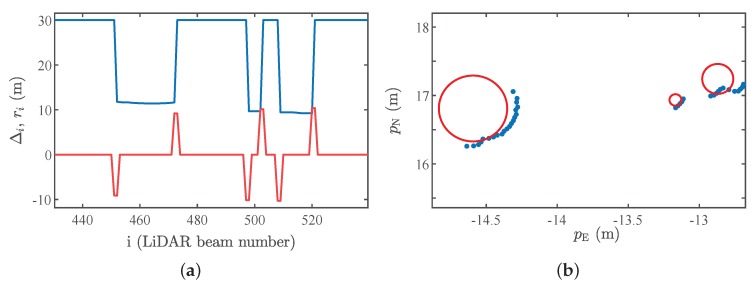
(**a**) Original laser scan in blue and the detected discontinuities in red. (**b**) Tree radius estimate. The blue dots represent the laser beams, and the tree trunks are represented by red circles.

Feature extraction is the first step towards accurate motion estimation. Considering that the operating environment is a forest, tree trunks seem to be natural choices for features. To detect the trunks in the LiDAR data we use three steps. First, the range measurements are constrained to minimum and maximum values; this is necessary to reduce the influence of noise in the measurements, which increases with distance, and to eliminate beams that hit parts of the vehicle. In the second step, laser scans are segmented using edge points, detected as discontinuities in the scan:

(1) $$\begin{array}{ccc} \Delta_{i} & = & \frac{r_{i+1}-r_{i-1}}{2}\;, \end{array}$$

where $r_{i}$ is the range measured by *i*th LiDAR beam for $i=2,\ldots ,n_{r}-1$, where $n_{r}$ is maximum number of beams. Note that such a segmentation strategy is widely used in the literature to generate clusters of laser data [26]. Figure 3a shows the original scan, represented by a sequence of range values $r_{i}$ and the detected discontinuities $\Delta_{i}$. To mitigate measurement noise, we only accept $\Delta_{i}\geq 0.1$ m. In addition, to compute the discontinuity $\Delta_{i}$, we use the range beams in positions i−1 and i+1, which diminishes the influence of outliers in the measurements. A tree trunk is probably found between the peaks down and up of the signal.

**Figure 4:**
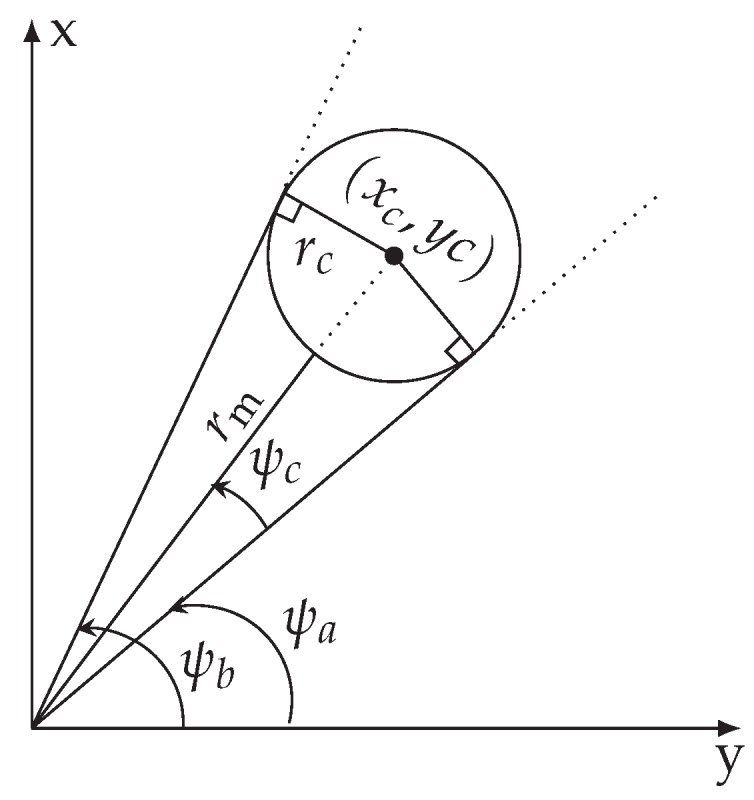
Procedure to compute the radius of tree trunk.

The third step of the feature extraction algorithm assumes that all trunks are cylindrical and estimates the radius $r_{c}$ of each trunk. Figure 4 illustrates the radius estimation method used in our work. This method follows the procedure proposed by the authors of [26], and computes the radius as

(2) $$\begin{array}{ccc} r_{c} & = & \frac{r_{m}\sin\left(\psi_{c}\right)}{1-\sin(\psi_{c})}, \end{array}$$

where $\psi_{c}=\left(\psi_{b}-\psi_{a}\right)/2$ is the angle of the beam that hits the center of the tree and $\psi_{a}$ and $\psi_{b}$ are the angles of the edges. Figure 3b shows some trees (red circles) estimated using this method. To eliminate possible wrong features, such as bushes, we only consider tree trunks with radius greater than 0.1 m and less than 1.5 m.

Before the estimation of the circles that model the trees, the range information from the LiDAR was transformed from the body coordinate frame to the NED coordinate frame. Thus, the positions of the centers of the circles are represented in NED. These centers are then considered as features and used in an Iterative Closest Points (ICP) algorithm [27]. This algorithm is used to establish the correspondence between the features just found with the ones found in a previous instant of time. Thus, for the set of features $D=\{d_{1},\;d_{2},\;\ldots ,\;d_{n_{d}}\}$ at time step *k* and $M=\{m_{1},\;m_{2},\;\ldots ,\;m_{n_{m}}\}$ at time step l<k, where $d_{i}$, $m_{j}\in R^{2}$ are centers of the fitted circles and $n_{d}$ and $n_{m}$ are the numbers of features extracted, the problem is to find an alignment; rotation, *R*; and translation, ρ that minimizes the distance between the two sets of points. This can be formulated as

(3) $$\begin{array}{c} \epsilon \left(R,\rho \right)=\sum_{i=1}^{n_{c}}∥Rd_{i}+\rho -m_{i}{∥}^{2}\;, \end{array}$$

(4) $$\begin{array}{c} \left(R^{*},\rho^{*}\right)=\underset{R,\rho }{\arg\;\min}\;\epsilon \left(R,\rho \right)\;, \end{array}$$

where ϵ(R,ρ) is called ICP metric fit error and $n_{c}$ is the number of common features between the current feature set D and the past feature set M. The closed-form solution for the least-squares problem of Equation (4) in 2D is given by [28]

$$\begin{array}{ccc} R^{*} & = & R(\psi )\;,\\ \rho^{*} & = & \bar{m}-R^{*}\;\bar{d}\;, \end{array}$$

where R(ψ) is a 2D rotation matrix that represents the vehicle rotation of angle ψ about the vertical axis, $\bar{m}≜\frac{1}{n_{c}}\sum_{i}^{n_{c}}m_{i}$, $\bar{d}≜\frac{1}{n_{c}}\sum_{i}^{n_{c}}d_{i}$, and

$$\psi =\arctan\left(\frac{S_{12}-S_{21}}{S_{11}+S_{22}}\right)\;,$$

with $S_{ij}$ being the element ij of the covariance matrix $S≜\frac{1}{n_{c}}\sum_{i}^{n_{c}}\left(d_{i}-\bar{d}\right){\left(m_{i}-\bar{m}\right)}^{T}$.

### 3.2. Mathematical Modeling

In this section, the stochastic models used in the state estimators are presented. First, we present the process model *f*, which is based on the kinematic equations of the aircraft. Then, we show the observation model *h*, which relates the measured output data *y* with the vehicle’s states *x*. The notation used through this section is listed at the end of the paper.

#### 3.2.1. Process Model

The temporal evolution of the vehicle dynamics is described by two sets of nonlinear first-order ordinary differential equations relative to a local NED coordinate frame.

The set of equations related to the position of the vehicle’s center of gravity, $p={\left[p_{N}\;p_{E}\;p_{D}\right]}^{T}\;\in \;R^{3}$, with respect to the NED coordinate frame, is given by

(5) $$\begin{array}{ccc} \dot{p}\left(t\right) & = & v(t). \end{array}$$

The time evolution of the linear velocity with respect to NED, $v={\left[v_{N}\;v_{E}\;v_{D}\right]}^{T}\in R^{3}$ is given by

(6) $$\begin{array}{ccc} \dot{v}\left(t\right) & = & R_{b}^{\mathrm{NED}}\left(e\right)a\left(t\right)+g, \end{array}$$

where $g={\left[0\;0\;g_{z}\right]}^{T}\;\in \;R^{3}$ is the gravity acceleration vector with $g_{z}=9.81\;$m/s${}^{2}$, and $R_{b}^{\mathrm{NED}}$ is the orthogonal rotation matrix that represents the rotation of the body coordinate frame with respect to the NED coordinate frame (the work by the authors of [29], p. 256). In this work, this rotation matrix is computed using the attitude provided by the AHRS. In systems for which this information is not provided, such attitude information needs to be estimated together with the other vehicle states. Also, it is important to mention that, for the case of high velocities, a term corresponding to the Coriolis effect may be added to Equation (6). In this work, we assume that the vehicle achieves small enough velocities.

Aiming at discrete-time state estimators, the continuous-time dynamic Equations (5) and (6) are discretized by integrating over time interval [(k−1)T,kT], where t=kT relates continuous time to the discrete index and T>0 is the sampling period. In this case, only the right-hand end point of [(k−1)T,kT], given by $x_{k}≜x\left(kT\right)$, is used. In this work, for simplicity, the Euler integration method is used for discretization (see the work by the authors of [30], p. 26).

Then, it is considered that the measured input vector $u_{k}={\left[a_{k}^{T}\;\;e_{k}^{T}\right]}^{T}\;\in \;R^{6}$ is corrupted by bias $\beta_{a,k}$ and random noise $q_{k}$, and are modeled as

(7) $$\begin{array}{ccc} a_{m,k} & = & a_{k}+\beta_{a,k}+q_{a,k}, \end{array}$$

(8) $$\begin{array}{ccc} e_{m,k} & = & e_{k}⊕q_{e,k}, \end{array}$$

where $\beta_{a,k}=\left[\beta_{a_{x}}\right.$$\beta_{a_{y}}$$\beta_{a_{z}}{]}^{T}\in R^{3}$ are the bias terms, $q_{a,k}∼N\left({\left[0\right]}_{3\times 1},\;Q_{a}\right)\;\in \;R^{3}$ is the acceleration noise vector, and $q_{e,k}∼N\left({\left[0\right]}_{3\times 1},\;Q_{e}\right)\;\in \;R^{3}$ is the orientation noise vector parameterized as “rotation vector”. The operator ⊕, as in the work by the authors of [21], maps the rotation vector $q_{e,k}$ to a quaternion and rotates $e_{k}$.

The accelerometer bias $\beta_{a,k}$ is modeled as a random-walk process:

(9) $$\begin{array}{ccc} \beta_{a,k} & = & \beta_{a,k-1}+q_{\beta ,k-1}\;, \end{array}$$

where $q_{\beta }∼N\left({\left[0\right]}_{3\times 1},\;Q_{\beta }\right)\in R^{3}$. The bias components are jointly estimated with vehicle states, yielding the “joint state vector” ${\overset{ˇ}{x}}_{k}\;\in \;R^{9}$, defined as

(10) $$\begin{array}{ccc} {\overset{ˇ}{x}}_{k} & ≜ & {\left[p_{k}^{T}\;\;v_{k}^{T}\;\;\beta_{a,k}^{T}\right]}^{T}\;. \end{array}$$

In our work, LiDAR-based odometry (LO) yields relative measurements, which means that it depends on past states. Therefore, state vector in Equation (10) is augmented with a “clone”, ${\overset{`}{p}}_{l}^{c}={\left[p_{N}^{c}\;p_{E}^{c}\right]}^{T}\in R^{2}$, of the position states projected in the xy-plane as estimated in time step l<k, ${\overset{`}{p}}_{l}={\left[p_{N}\;p_{E}\right]}^{T}\in R^{2}$. Here, the term “clone” (represented by the superscript “c”) is used to define a simple and exact copy of a past state, as is done by the authors of [14]. After a new LO measurement is obtained and used to correct the system estimates (see Section 3.3.1), the cloned states are updated with the newest estimate of ${\overset{`}{p}}_{k}$. The equation that describes the evolution of the cloned states with respect to time is given by

(11) $$\begin{array}{ccc} {\overset{`}{p}}_{k}^{c} & = & {\overset{`}{p}}_{k-1}^{c}. \end{array}$$

Notice that there is no noise in this model, indicating that the cloned states remain the same until they are replaced by a new clone. We define the augmented state vector $x_{k}\in R^{11}$ as

(12) $$\begin{array}{ccc} x_{k} & ≜ & {\left[{\overset{ˇ}{x}}_{k}^{T}\;{\left({\overset{`}{p}}_{k}^{c}\right)}^{T}\right]}^{T}\;. \end{array}$$

The discretized version of Equations (5) and (6) together with Equations (9) and (11) compose the “process model” of the vehicle, which can be compactly recast as

(13) $$\begin{array}{ccc} x_{k} & = & f\left(x_{k-1},\;u_{k-1},\;q_{k-1},\;k-1\right)\;. \end{array}$$

#### 3.2.2. Observation Model

The observation model relates the components of the state vector $x_{k}$ with the measured output variables $y_{k}\;\in \;R^{8}$ is given by

(14) $$\begin{array}{c} y_{k}≜{\left[p_{m,k}\;v_{m,k}\;\delta_{m,k}\right]}^{T}. \end{array}$$

Global position and velocity are given by the GNSS system and are modeled as

(15) $$\begin{array}{ccc} p_{m,k} & = & p_{k}+r_{p,k}\;, \end{array}$$

(16) $$\begin{array}{ccc} v_{m,k} & = & v_{k}+r_{v,k}\;, \end{array}$$

where $r_{p,k}∼N\left({\left[0\right]}_{3\times 1},\;R_{p}\right)\in R^{3}$ and $r_{v,k}∼N\left({\left[0\right]}_{3\times 1},\;R_{v}\right)\in R^{3}$ are the position and velocity noises, respectively.

LiDAR-odometry gives incremental displacement, $\delta_{m,k}\in R^{2}$, in the xy-plane, which means that the measurement depends both on the current ${\overset{`}{p}}_{k}$ and the past states, stored as clone ${\overset{`}{p}}_{k}^{c}$ (see Equation (12)). The augmentation of the state vector with a copy (clone) of the past state is the approach known as stochastic cloning [14]. Then, assuming that the state vector is augmented with the position states ${\overset{`}{p}}_{k}^{c}$, the relative measurement model is given by

(17) $$\begin{array}{ccc} \delta_{m,k} & = & {\overset{`}{p}}_{k}-{\overset{`}{p}}_{k}^{c}+r_{\delta ,k}, \end{array}$$

where $r_{\delta ,k}∼N\left({\left[0\right]}_{2\times 1},\;R_{\delta p}\right)\in R^{2}$.

The complete observation model may be written as

(18) $$\begin{array}{ccc} y_{k} & = & h\left(x_{k},\;r_{k},\;k\right), \end{array}$$

where *h* is a function of random noise $r_{k}$ and the current $x_{k}$ states given by Equations (15)–(17).

### 3.3. Nonlinear State Estimator

In this paper, we assume that the dynamic system is modeled by the nonlinear state-space Equations (13) and (18), in which ∀k≥1 and the known data are the measured output $y_{k}$ and input $u_{k-1}$. It is also assumed that process noise, $q_{k-1}\in R^{n_{q}}$, and output measured noise, $r_{k}\in R^{n_{r}}$, are mutually independents with covariance matrices of $Q_{k-1}\in R^{n_{q}\times n_{q}}$ and $R_{k}\in R^{n_{r}\times n_{r}}$, respectively. Under these assumptions, the state estimation problem aims at providing approximations for the mean ${\hat{x}}_{k}=E\left[x_{k}\right]$ and covariance $P_{k}^{xx}=E\left[\left(x_{k}-{\hat{x}}_{k}\right){\left(x_{k}-{\hat{x}}_{k}\right)}^{T}\right]$ that characterize the *a posteriori* probability density function (PDF) $\rho (x_{k}|y_{1:k})$.

Due to the nonlinear characteristics of the model, we use, as basis to our approach, the unscented Kalman filter (UKF) [31]. In the standard form of the UKF, two problems arise: (i) due to the stochastic cloning approach, the covariance matrix $P_{k}^{xx}$ may become negative semidefinite [8], which is inconsistent with its definition, and (ii) the output measured noise $r_{k}$ can have time-varying statistical properties, which can degrade the estimates. Regarding (i), a modification based on statistical linear regression (SLR), similar to what was used by Shen et al. [8], is shown in Section 3.3.1. The solution to (ii) is our core contribution. We consider two events that may change the statistical properties of measured noise: sudden jumps and slow varying error in the measurements. The rejection of these perturbations are addressed in Section 3.3.2 and Section 3.3.3.

#### 3.3.1. Unscented Kalman Filter for Absolute and Relative Measurements

Let the process noise be partitioned as $q_{k-1}≜{\left[q_{1,k-1}^{T}\;\;q_{2,k-1}^{T}\right]}^{T}\;\in \;R^{15}$ with covariance matrix $Q_{k-1}≜\mathrm{diag}\left(Q_{1,k-1},\;Q_{2,k-1}\right)\;\in \;R^{15\times 15}$, where $q_{1,k-1}\;\in \;R^{6}$ is the multiplicative noise related to the state vector and $q_{2,k-1}\;\in \;R^{9}$ is the additive partition of noise. To improve the numerical stability of the filter, additive noise is considered for all states [32]. Henceforth, the notation ${\hat{x}}_{k|k-1}$ indicates an estimate of $x_{k}$ at time *k* based on information available up to and including time k−1. Likewise, ${\hat{x}}_{k}$ indicates an estimate of $x_{k}$ at time *k* based on information available up to and including time *k*.

Given these definitions, the modified UKF forecast step is given by

(19) $$\begin{array}{ccc} \left({\hat{\overset{ˇ}{x}}}_{k|k-1},{\tilde{P}}_{k|k-1}^{\overset{ˇ}{x}\overset{ˇ}{x}},P_{k|k-1}^{\overset{ˇ}{x}\bar{\overset{ˇ}{x}}}\right) & = & \mathrm{UT}\left({\hat{x}}_{k-1}^{a},P_{k-1}^{x^{a}x^{a}},u_{k-1},f\right),\; \end{array}$$

(20) $$\begin{array}{ccc} P_{k|k-1}^{\overset{ˇ}{x}\overset{ˇ}{x}} & = & {\tilde{P}}_{k|k-1}^{\overset{ˇ}{x}\overset{ˇ}{x}}+Q_{2,k-1}, \end{array}$$

in which, UT(·) is the unscented transform function, as defined in the work by the authors of [22], $x_{k-1}^{a}\in R^{15}$ and $P_{k-1}^{x^{a}x^{a}}\in R^{15\times 15}$ are respectively the augmented state vector and its corresponding covariance matrix, given by

$$\begin{array}{ccc} x_{k-1}^{a} & ≜ & {\left[\begin{array}{cc} {\overset{ˇ}{x}}_{k-1}^{T} & q_{1,k-1}^{T} \end{array}\right]}^{T}\;,\\ P_{k-1}^{x^{a}x^{a}} & ≜ & \left[\begin{array}{cc} P_{k-1}^{\overset{ˇ}{x}\overset{ˇ}{x}} & {\left[0\right]}_{9\times 6}\\ {\left[0\right]}_{6\times 9} & Q_{1,k-1} \end{array}\right]\;.\; \end{array}$$

Recall that, in the previous step, the cloned states are not part of the augmented state vector. Then, the propagated cloned state and covariance is computed as

(21) $$\begin{array}{ccc} {\hat{x}}_{k|k-1} & = & {\left[\begin{array}{cc} {\hat{\overset{ˇ}{x}}}_{k|k-1}^{T} & {\left({\hat{\overset{`}{p}}}_{k-1}^{c}\right)}^{T} \end{array}\right]}^{T}, \end{array}$$

(22) $$\begin{array}{ccc} P_{k|k-1}^{xx} & = & \left[\begin{array}{cc} P_{k|k-1}^{\overset{ˇ}{x}\overset{ˇ}{x}} & F_{k}P_{k-1}^{\overset{`}{p}\overset{ˇ}{x}}\\ P_{k-1}^{\overset{ˇ}{x}\overset{`}{p}}F_{k}^{T} & P_{k-1}^{\overset{`}{p}\overset{`}{p}} \end{array}\right], \end{array}$$

where $F_{k}$ is computed as

(23) $$\begin{array}{ccc} F_{k} & = & {\left(P_{k|k-1}^{\overset{ˇ}{x}\bar{\overset{ˇ}{x}}}\right)}^{T}{\left(P_{k-1}^{\overset{ˇ}{x}\overset{ˇ}{x}}\right)}^{-1}. \end{array}$$

The state estimate and error covariance matrix are updated using information from $y_{k}$ in the data assimilation step, given by

(24) $$\begin{array}{ccc} \left({\hat{y}}_{k|k-1},{\tilde{P}}_{k|k-1}^{yy},P_{k|k-1}^{xy}\right) & = & \mathrm{UT}\left({\hat{x}}_{k|k-1},P_{k|k-1}^{xx},0,h\right)\;\;,\; \end{array}$$

(25) $$\begin{array}{ccc} P_{k|k-1}^{yy} & = & {\tilde{P}}_{k|k-1}^{yy}+R_{k}, \end{array}$$

(26) $$\begin{array}{ccc} \nu_{k} & = & y_{k}-{\hat{y}}_{k|k-1}, \end{array}$$

where $\nu_{k}$ is the innovation.

(27) $$\begin{array}{ccc} K_{k} & = & P_{k|k-1}^{xy}{\left(P_{k|k-1}^{yy}\right)}^{-1}, \end{array}$$

(28) $$\begin{array}{ccc} {\hat{x}}_{k} & = & {\hat{x}}_{k|k-1}+K_{k}\nu_{k}, \end{array}$$

(29) $$\begin{array}{ccc} P_{k}^{xx} & = & P_{k|k-1}^{xx}-K_{k}P_{k|k-1}^{yy}K_{k}^{T}. \end{array}$$

After measurement update, the cloned states ${\hat{\overset{`}{p}}}_{k}^{c}$ are replaced with a new copy of current states ${\hat{\overset{`}{p}}}_{k}$ and a new covariance matrix $P_{k}^{xx}$ is computed, as per the authors of [9],

(30) $$\begin{array}{ccc} P_{k}^{xx} & = & C\left(P_{k}^{\overset{ˇ}{x}\overset{ˇ}{x}}\right)C^{T},\\ C & = & \left[\begin{array}{ccc} I_{3\times 3} & {\left[0\right]}_{3\times 3} & {\left[0\right]}_{3\times 3}\\ {\left[0\right]}_{3\times 3} & I_{3\times 3} & {\left[0\right]}_{3\times 3}\\ {\left[0\right]}_{3\times 3} & {\left[0\right]}_{3\times 3} & I_{3\times 3}\\ \left[\begin{array}{cc} I_{2\times 2} & {\left[0\right]}_{2\times 1} \end{array}\right] & {\left[0\right]}_{2\times 3} & {\left[0\right]}_{2\times 3} \end{array}\right]\;,\; \end{array}$$

where ${\left[0\right]}_{n\times n}$ and $I_{n\times n}$ are zero and identity matrices with *n* by *n* elements, respectively. Notice that the operations with the cloned states are performed only when new relative measurement is available.

#### 3.3.2. Adaptive Measurement Covariance Matrix

The uncertainty of the measurements in the UKF is represented by the covariance matrix $R_{k}$. This is usually a predefined parameter, which remains constant. However, as previously commented, the measurement uncertainties can be time-varying. We then propose the use of innovation $\nu_{k}$ to tune the measurement covariance matrix online through the covariance matching (CM) approach [16].

Based on the assumption that the observation covariance matrix $R_{k}$ is constant during a sliding sampling window with finite length *N*, the basic idea of CM is to make the innovation $\nu_{k}$ consistent with its covariance $E\left[\nu_{k}\nu_{k}^{T}\right]≜P_{k|k-1}^{yy}$. The covariance of $\nu_{k}$ is estimated as based on the last *N* innovation samples as

(31) $$\begin{array}{c} E\left[\nu_{k}\nu_{k}^{T}\right]\approx \frac{1}{N}\sum_{j=k-N+1}^{k}\nu_{j}\nu_{j}^{T}. \end{array}$$

Notice that the UKF (see Equation (25)) approximates the covariance by $E\left[\nu_{k}\nu_{k}^{T}\right]≜{\tilde{P}}_{k|k-1}^{yy}+R_{k}$. Then, $R_{k}$ can be estimated as

(32) $$\begin{array}{ccc} {\hat{R}}_{k} & = & \frac{1}{N}\sum_{j=k-N+1}^{k}\nu_{j}\nu_{j}^{T}-{\tilde{P}}_{k|k-1}^{yy}. \end{array}$$

To avoid negative values due the subtraction operation in Equation (32), the following treatment is performed,

(33) $$\begin{array}{ccc} {\hat{R}}_{k} & = & \max\left(\frac{1}{N}\sum_{j=k-N+1}^{k}\nu_{j}\nu_{j}^{T}-{\tilde{P}}_{k|k-1}^{yy},R_{0}\right)\;,\; \end{array}$$

where $R_{0}$ is a lower threshold given by the nominal measurement-noise covariance, which may be empirically determined, and max(A,B) returns a diagonal matrix with the max elements taken from the diagonal of *A* and *B*.

#### 3.3.3. Outlier Rejection

Outliers are spurious data that contaminate the statistical distribution. The contaminated measurements may deviate significantly from the “normal” observations, thus directly reflecting in the innovation value $\nu_{k}$, and, consequently, in the covariance estimated by CM.

To minimize the influence of outliers, a particular method is to judge each element of the innovation with a $\chi^{2}$-test [33]. Thus, for the *i*th element of the innovation vector, the normalized innovation squared can be computed as

(34) $$\begin{array}{ccc} \epsilon_{k,i}^{\nu } & = & \frac{\nu_{k,i}^{2}}{P_{k-1|k-2,ii}^{yy}}. \end{array}$$

Under the linear-Gaussian assumption, the PDF of $\epsilon_{k,i}^{\nu }∼\chi_{1}^{2}$ is a chi-square distribution with one degree of freedom. Then, for a significance level $\alpha \in \left[0,1\right]$, the probability of a “normal” measurement is $p\left(\epsilon_{k,i}^{\nu }\leq \zeta \right)=1-\alpha ,$ where ζ∈R is the a value taken from the chi-square cumulative distribution function. Thus, we can replaced the current innovation as

(35) $$\begin{array}{c} {\hat{\nu }}_{k}=\min\left(1,\frac{\zeta }{\epsilon_{k,i}^{\nu }}\right)\nu_{k}\;, \end{array}$$

where $\min\left(A,B\right)$ is a function that returns the minimum value between *A* and *B*.

One might ask, why we weaken the innovation instead of just drop the abnormal measurement completely? The reason is that, besides detecting the presence of abnormal behaviors, statistical procedures, such as (35), can still extract some remained information from the innovation. For instance, we could receive a measurement with a scale error. In such a case, the outlier rejection procedure can alter the measurement scale, eliminating the wrong information.

#### 3.3.4. Robust Adaptive Unscented Kalman Filter

By combining Equations (33) and (35) with Equations (19)–(30), we then obtain a three step recursive algorithm that we call Robust Adaptive Unscented Kalman Filter (RAUKF) (see Figure 2). The first step of this algorithm is the forecast step, which is given by Equations (19)–(22). The second step is the robust noise estimation given by Equations (24), (26), (35), (33), and (25). The third and last step is the data assimilation step are given by Equations (27)–(30).

Observe that the measurement sampling rate may be different for each sensor, yielding a different measurement vector at each time instant. For the case where only GNSS measurements are available, it is not necessary to compute Equation (30) and replace the cloned states with a new copy. Thus, the data assimilation step is only given by Equations (27)–(29). When GNSS is not available but LO is still available, the measurement output variables vector $y_{k}$ in Equation (14) will contain only altitude, given by the vehicle’s barometer, and relative motion information, $\delta_{m,k}$. The data assimilation step will be executed by Equations (27)–(30).

## 4. Motion Control

We divide our motion control approach in three steps. In the first step, we process the data from the LiDAR and construct a local map for collision avoidance. In the second step, a probabilistic motion planner uses this map to compute the vehicle path, and, finally, a velocity controller drives the vehicle through the planned path. We start by describing our mapping approach, which is one of the contributions of this paper.

### 4.1. Local Mapping for Collision Avoidance

Probabilistic planners usually do not require a map of the workspace, but instead they will need a function that returns as fast as possible if a given configuration is in collision or not. The literature has shown that the time spent to construct search trees by probabilistic planners such as RRT*, is 99% concentrated in checking if the random configurations are in collision [34]. In this section, we present an efficient mapping strategy that facilitates collision checking by the planner.

By using the MAV’s position and orientation estimated by RAUKF, the first step of our local mapping methodology is to transform, using homogenous transformation matrices, the LiDAR points represented with respect to the vehicle coordinate frame into the NED fixed frame. Next, we define analytically all the obstacles found.

**Figure 5:**
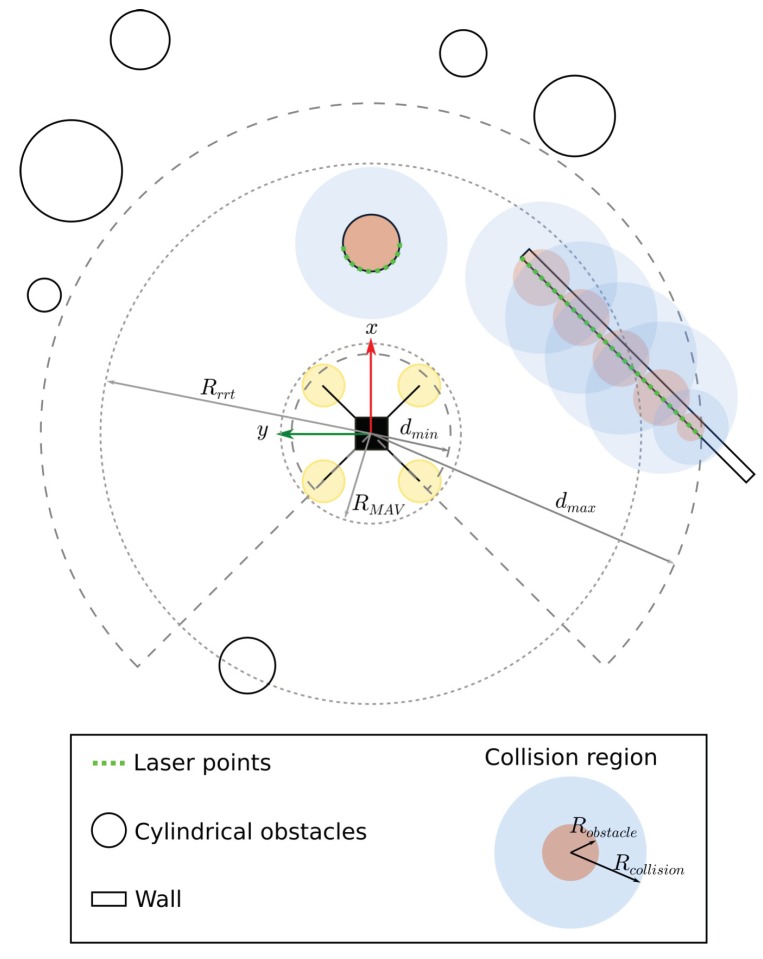
MAV in a environment with 7 cylindrical trees and and a wall. Only the obstacles between $d_{\min}$ and $d_{\max}$ are used to build the local map. Obstacles outside the circle of radius $R_{\mathrm{rrt}}$ are not used during path planning.

With the method described in Section 3.1, we process the LiDAR data and approximate each obstacle classified as trees by cylinders (circles in the plane). For the sake of simplicity, those obstacles that are not classified as trees, i.e., obstacles that have a radius is too big or too small, are also approximated by circles. For this case, point sequences of up to *n* points are grouped, and each group is represented by a circle of diameter given by the distance between the first and the last point in the sequence. To guarantee safety during path planning, the radius of all circles, including trees and non-trees, is increased by the radius of the robot, $R_{\mathrm{MAV}}$, plus a small safety amount $R_{s}$, which results in radius $R_{\mathrm{collision}}$. Figure 5 illustrates this approach.

Figure 5 also shows other two strategies that we use to speed up the collision check by the planner. First, the planner (in our implementation RRT*) only searches for paths in a finite horizon determined by a circle of radius $R_{\mathrm{rrt}}$ centered in the current vehicle position. Second, we define minimum and maximum distances, $d_{\min}$ and $d_{\max}$, respectively, in which we consider that LiDAR points are obstacles.

**Figure 6:**
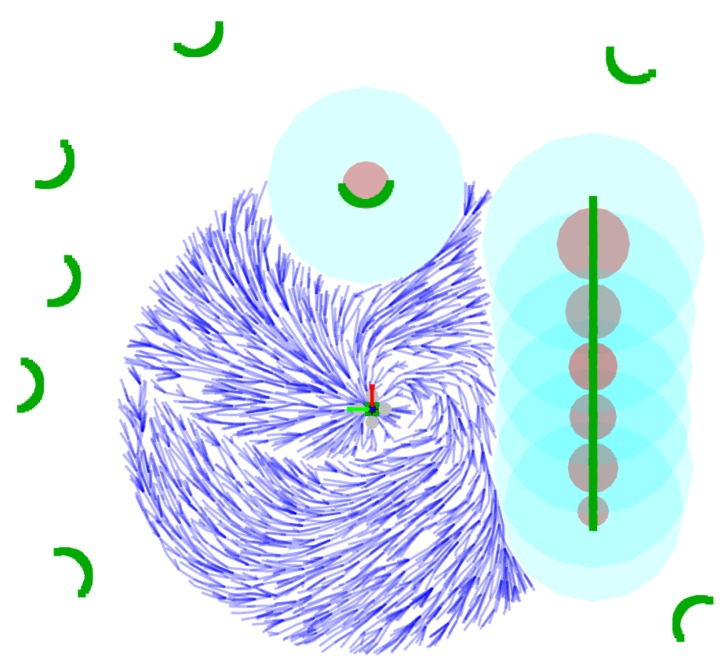
Simulation where a micro air vehicle (MAV) is flying in a forest with several trees and a long obstacle, such as a wall. LiDAR data is shown in green. If the obstacle is within the region of interest, it is approximated by a circle (brown disk). The light-blue region shows the expanded obstacle and the dark-blue graph represents the planner’s search tree.

With these strategies, each collision checking operation is reduced by testing if a point is inside a small number of circles, which are approximately the same number of obstacles close to the drone (see Figure 6). In the worst case, for instance, if n=50 and the number of laser points is 1041, the number of circles would be only 21.

### 4.2. Path Planning

**Figure 7:**
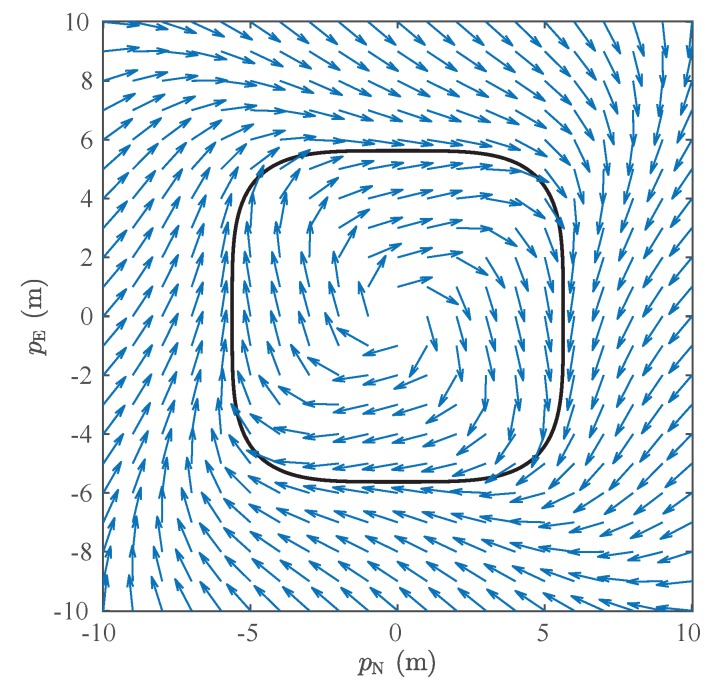
Vector field (blue arrows) used in this work to define a periodic MAV’s surveillance and monitoring task. By following the vector field, the vehicle will converge and circulate the solid black path.

The path planning approach used in this paper to navigate in forests is based on the proposed by Pereira et al. [23]. This is a two-level planner where the higher level specifies, using an artificial vector field, the task of the MAV. If the drone must execute a periodic surveillance or monitoring task, for example, we use a vector field that forces the vehicle to converge to and circulate along a closed curve. A methodology to generate such a vector field was proposed by Gonçalves et al. [24]. Figure 7 shows an example of a vector field, $\vartheta (p_{E},p_{N})$, for circulation of a curve parameterized by $\alpha \left(p_{E},p_{N}\right)=p_{N}^{4}+p_{E}^{4}-1000=0$. Notice that this vector field can be constructed by the composition of vectors normal to the curve and vectors tangent to the curve.

As the vector field does not consider the position of the trees and other obstacles in the forest, in a lower level, our strategy uses a local planner that runs for each update of the local map constructed with LiDAR information (Section 4.1). The local planner adopted in this work is RRT* [25], which is usually a global planner, but here, as it runs in a small region of the workspace, it does not lead the robot to any global goal position. In fact, the strategy proposed by the authors of [23] does not require any goal position, and the direction of the movement is determined by the vector field through the minimization of the following cost functional by RRT*:

(36) $$\begin{array}{c} F\left[\xi ,\vartheta \right]=\int_{0}^{1}\left(1-\;\frac{\;\xi^{\prime }\left(s\right)}{∥\xi^{\prime }\left(s\right)∥}\cdot \frac{\vartheta \left(\xi (s)\right)}{∥\vartheta (\xi (s))∥}\right)\;∥\xi^{\prime }\left(s\right)∥\;ds\;, \end{array}$$

where ξ(s) is the MAV’s path, $\vartheta \left(p\right),p\in R^{2}$ is the vector field, the upper comma stands for the derivative with respect to the spacial parameterization variable *s* of the path ξ, operator ∥·∥ represents the Euclidean norm, and the center dot is the scalar product. Notice that this functional is a function of both the length of the path and on how “close” the path is from the vector field. In fact, F[ξ,ϑ] is greater or equal to zero, and is zero if and only if the path is parallel to the field ($\xi^{\prime }\left(s\right)=\gamma \;\vartheta (\xi (s))$, for γ>0).

To avoid discontinuity in the MAV’s global path, it is important to guarantee that the initial position of each local path computed by RRT* is the final position of the previous one. To guarantee that, each local path is integrated (simulated) for the time of planning $t_{\mathrm{loop}}$ to find point $p_{\mathrm{end}}$. Although the vehicle follows the current path, a new instance of RRT* thus compute the path that starts at $p_{\mathrm{end}}$. The next section shows how the computed path can be followed by the drone.

### 4.3. Path Following

As a result of path planning, a path is defined as a sequence of points in $R^{3}$. As we assumed a 2D vector field, the coordinate *z* of this path is constant and specifies the height or altitude of the flight. To make the MAV to follow the path, we define a second vector field, this time in 3D, using the methodology proposed in the work by the authors of [24]. The resultant field is considered to be a velocity field that has an attractive component that is orthogonal to the path and another component parallel to path, which is responsible by the robot’s longitudinal velocity.

The computed path does not define a orientation to the vehicle. However, besides following the path, we use a heading controller that keeps the front of the robot pointing to the direction of the path. This is done to guarantee that the planar LiDAR, which usually does not have a $360^{\circ }$ field of view, is able to detect obstacles in front of the MAV.

## 5. Experimental Results

**Figure 8:**
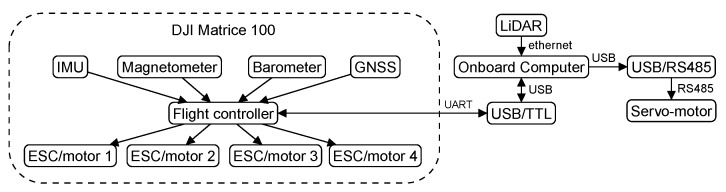
Experimental setup diagram.

This section presents an experiment with the customized commercial aerial vehicle shown in Figure 1. Our DJI Matrice 100 quadrotor, which is commercialized with built-in AHRS and GNSS, was equipped with a Hokuyo UTM-30LX-EW planar LiDAR, which has a scanning frequency of 40 Hz. The LiDAR was mounted on a servo-motor, which allowed us to compensate for the vehicle’s roll angle. As we do not have a way to compensate for the vehicle’s pitch angle, the LiDAR was calibrated so that range data is easily transformed to the body coordinate frame, and from the body frame to NED. Once represented in NED, laser points that are more than 1m from the current height of the vehicle are filtered out, so that the vehicle only detects obstacles that are, approximately, on its motion plane. AHRS and GNSS are, in fact, a DJI proprietary navigation solution that runs at the low-level hardware and delivers attitude, global position, and velocity information in a fairly high frequency, that is, 100 Hz for attitude and 50 Hz for position. Table 1 shows the main hardware components of our setup, and Figure 8 shows how these components are interconnected.

**Figure 9:**
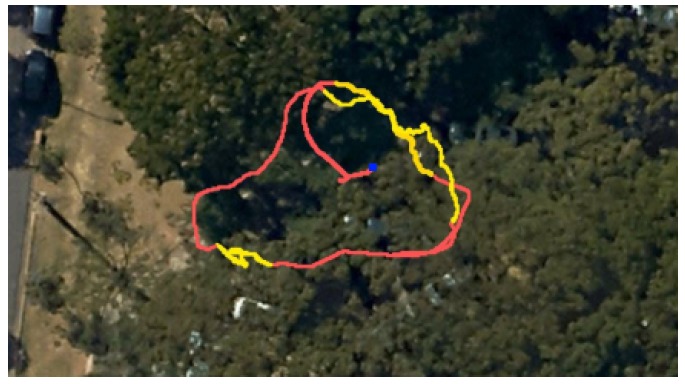
Satellite view of the forest where our experiments were executed. Image provided by Google Maps. The path estimated by RAUKF is shown in red whenever AHRS, LO, and GNSS were available and in yellow if only AHRS and LO were available. The vehicle’s starting point is represented by the blue square.

In our navigation system, the 100 Hz filter output is used to feedback the motion control system. The complete system runs on the on-board computer Odroid XU4 with an octa-core ARM processor, 2 GB of RAM, running Ubuntu Mate 16.04. The entire navigation system was developed in C++ using the Robot Operating System (ROS) as middleware. Synchronized data from DJI’s navigation solution are then provided as ROS topics. LiDAR scans are also provided by a ROS topic, and, upon availability, are used in the data assimilation step of our filter and to construct a local map. The experiment was performed in a forest environment in the main campus of UFMG, where the vehicle was commanded to fly at 1 m/s. Figure 9 shows a satellite view of the environment and vehicle path as estimated by RAUKF. In this figure, GNSS was not available in the yellow parts of the path. In what follows we discuss the main characteristics and behavior of the proposed navigation system during this experiment. Note that, although we only present a single experiment in this paper, our system was tested before in similar situations. Other results of our localization system working alone can be seen in our previous conference paper [22].

### 5.1. Localization System

Due to tree canopies, although the vehicle is flying inside the forest, the GNSS signal can be damaged. As we do not use raw GNSS data, but the result of DJI’s positioning solution, we could not detect any damage in the vehicle’s position and velocity information during our experiment. Therefore, to assess the robustness of our navigation system during GNSS failures, we artificially blocked the GNSS signal in three periods of time, what was done via software while the vehicle flew autonomously.

**Figure 10:**
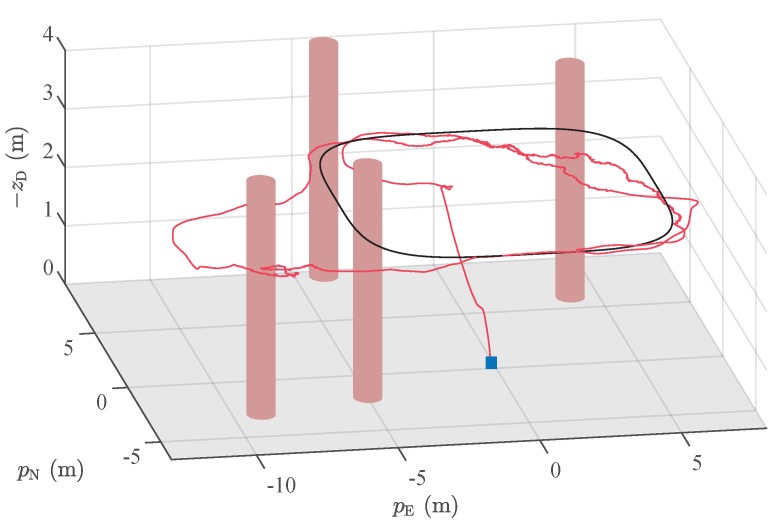
Three-dimensional path estimated by Robust Adaptive Unscented Kalman Filter (RAUKF) (red) for the target path represented by the black curve. The vehicle starting point is represented by the blue square. Observe that the estimated path deviates from the target one due to presence of trees, represented by brown cylinders. Note that, as the diameter and position of the trees are not known, such cylinders are approximations of the actual forest and are used for visualization only.

Figure 10 shows the target surveillance path (black curve) and RAUKF estimates (red curve) in 3D. We observe that our system was able to combine GNSS and relative LO measurements adequately. In addition, even in the absence of global measurements, RAUKF estimates position and velocity, allowing vehicle motion control. Notice that the target curve was not followed accurately, which is expected, as the initial plan did not considered the obstacles in the environment.

**Figure 11:**
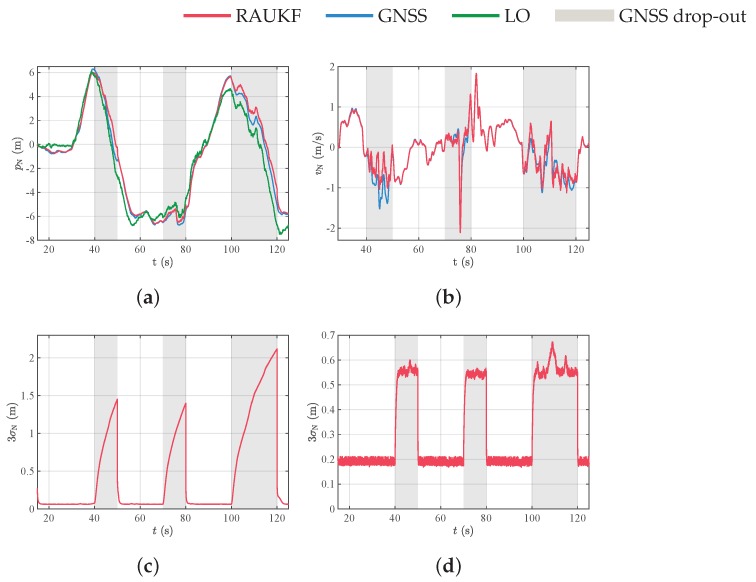
State estimation results. (**a**) Position and (**b**) velocity in the north direction; (**c**,**d**) three standard deviation $\sigma_{N}=\sqrt{P_{k,ii}^{xx}}$ of position and velocity estimates in the north direction.

**Figure 12:**
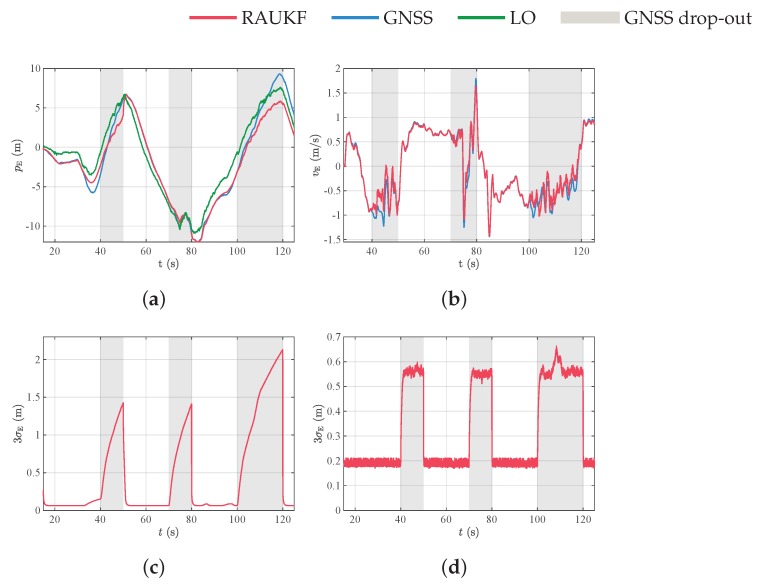
State estimation results. (**a**) Position and (**b**) velocity in the East direction; (**c**,**d**) three standard deviation $\sigma_{E}=\sqrt{P_{k,ii}^{xx}}$ of position and velocity estimates in the east direction.

Figure 11a,b and Figure 12a,b show the position and velocity of the MAV in the north and east directions, respectively. Although we did not use GNSS signal in the navigation system for some time intervals, for comparison purposes, we show all the measurements, including GNSS. The blockage periods of GNSS signal are delimited by gray-shaded regions. We observe that RAUKF position and velocity estimates tends to converge to GNSS position and velocity measurements. This behavior is expected, once GNSS is an absolute measurement, which is not the case of the LO. For the data in Figure 11a and Figure 12a, we also computed the Root-Mean-Square-Error (RMSE) of the position estimate in relation to GNSS in the periods where GNSS was artificially blocked and was not used in our filter. In these periods, GNSS measurements were then considered as ground truth information. The RMSE values were 0.65m and 1.5m for north and east directions, respectively.

Figure 11c,d and Figure 12c,d show the three standard deviations of position and velocity estimates in the north and east directions, respectively. Note that, whenever only LO relative measurements are used in the data assimilation step, the uncertainty grows unbounded, reflecting the error integration effect and non-observability of global position. On the other hand, due to relative measurements, the velocity uncertainty is bounded, meaning that the velocity is observable.

### 5.2. Motion Control System

**Figure 13:**
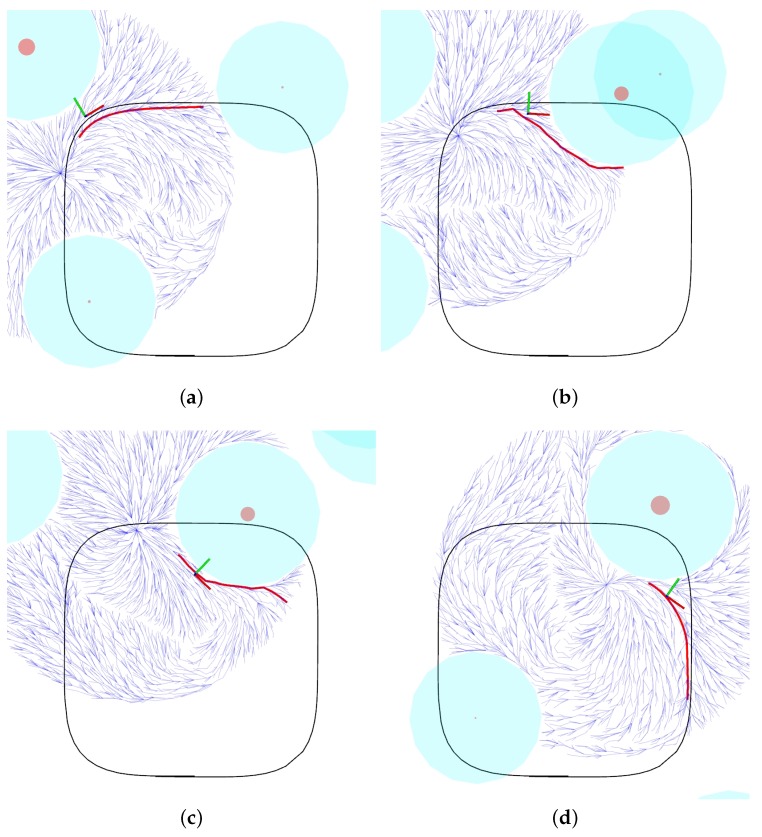
Four snapshots of our experiment detailing the behavior of the motion planner. The black line represents the target curve specified by the vector field. From the robot’s estimated position, the RRT* tree (in blue) grows inside a circle of radius $R_{rrt}$. (**a**) No obstacles are detected and the vehicle converges to the target curve. (**b**,**c**) An obstacle (brown cylinder) is detected and its collision region (light blue cylinder) is estimated. The planner then computed the path (in red) outside the collision region. (**d**) After avoiding the obstacle, the MAV is able to follow the target curve. Observe that the local map, including number, position, and size of the obstacles, change along the experiment. This happens due to changes in the vehicle orientation and noise in the LiDAR data.

**Figure 14:**
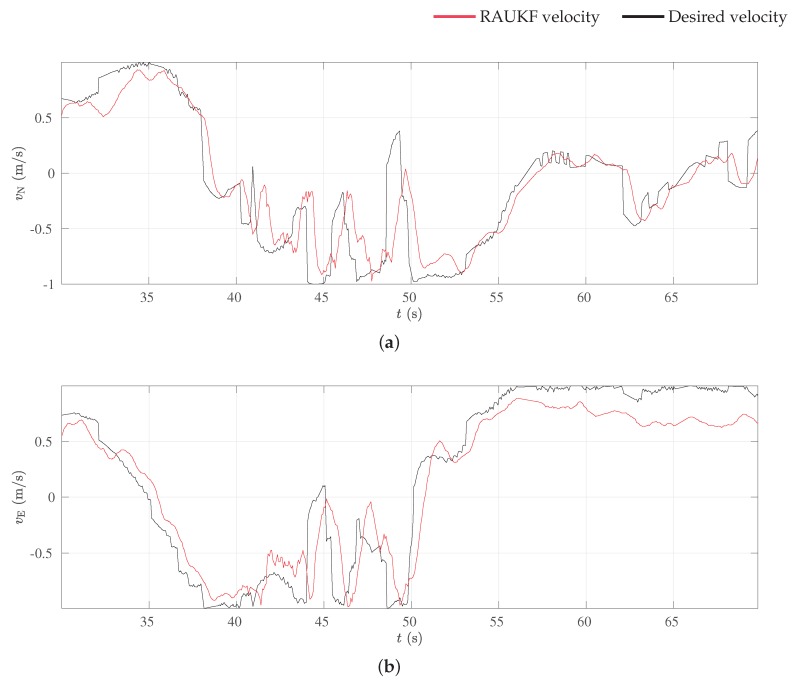
Attitude controller results. (**a**) Velocity in the north direction and (**b**) velocity in the east direction.

In the experiment, the quadrotor flied autonomously among the trees. While Figure 10 shows the MAV’s global path, Figure 13 shows four snapshots with the detailed behavior of the path planner, and Figure 14 shows planned and measured velocities for part of the experiment. For the data in Figure 14, the RMSE errors of the controller were 0.26m/s and 0.27m/s for north and east velocities, respectively. Notice in Figure 10 that, besides these control errors, the robot’s flight path converges to the specified curve whenever this is possible and deviate from it the presence of trees. Whenever the laser detects trees in the environment, these obstacles are added to the local map, allowing the local planner to compute a deviation path, necessary to avoid the tree. In this experiment, the desired task is represented by a closed surveillance path. The MAV follows the path for several laps until it is commanded to stop or its battery level becomes very low.

## 6. Conclusions

This paper presented a complete navigation solution for unmanned aerial vehicles navigating inside a forest. The proposed solution is based on two main systems, called localization and motion control. The localization algorithm combines LiDAR-based odometry and GNSS and AHRS information using a robust adaptive sensor fusion algorithm (RAUKF) based on the UKF. LiDAR-based odometry relies on the fact the trees are easily identified with a laser scan. It is important to mention that tree detection highly increased the efficiency of both localization and motion planning methods, allowing the system to run in a simple on-board hardware.

Our motion control approach is based on the combination of a vector field with and optimal planner for obstacle avoidance. This makes easier to the user to plan the main task of vehicle, which will be the simple definition of a curve in the space, letting obstacle avoidance to the MAV.

The experiment presented in this paper illustrated that the sensor fusion algorithm was able to adequately combine global and relative measurements. RAUKF was also able to deal with some abnormal information, both in relative and global measurements. By this experiment, we also observed that the vehicle was able to perform its surveillance task, although its actual path deviated from the target one, which is expected, as the target path does not considered obstacles.

In future, we plan to expand this work by using a 3D laser range finder, which will allow to plan the vehicle’s motion in all workspace dimensions. The 3D range finder can be also used to estimate the MAV’s orientation, which can be used in the data assimilation step of RAUKF. Finally, we also plan to include an autonomous landing system, which by now, is done by a human operator.

## Figures and Tables

**Table 1:** MAV components.

Component	Hardware
Quadrotor	DJI Matrice 100 with IMU, barometer, magnetometer and GNSS sensors
Flight controller	DJI N1
ESC/motor	DJI E Series 620D / DJI 3510
Onboard computer	Odroid XU4 with an octa-core ARM processor, 2 GB of RAM,
	running Ubuntu Mate 16.04
LiDAR	Hokuyo UTM-30LX-EW, 40 Hz, 30 m, $270^{\circ }$ scanning range
Servo-motor	Dynamixel MX-106R
USB/RS485 adapter	USB2Dynamixel
USB/TTL adapter	D-SUN, USB to TTL, CP2102

## Notation

The following notations are used in this manuscript:

$p={\left[p_{N}\;p_{E}\;p_{D}\right]}^{T}$	position with respect to the NED coordinate frame.
$v={\left[v_{N}\;v_{E}\;v_{D}\right]}^{T}$	velocity with respect to NED coordinate frame.
$g={\left[0\;0\;g_{z}\right]}^{T}$	gravity acceleration vector, where $g_{z}=9.81$ m/s${}^{2}$.
$R_{b}^{\mathrm{NED}}$	rotation matrix between the body coordinate frame and the NED coordinate frame.
$u_{k}$	input vector.
$a_{m,k}$	accelerometer measurement.
$e_{m,k}$	orientation measurement in unit quaternion.
$\beta_{a,k}$	accelerometer bias.
$q_{a,k}$	acceleration measurement noise.
$q_{\beta ,k}$	random walk noise.
$q_{e,k}$	orientation measurement noise.
${\overset{`}{p}}_{k}^{c}$	cloned state vector.
$x_{k}$	state vector.
$y_{k}$	output measurement.
$p_{m,k}$	position measurement given by GNSS.
$v_{m,k}$	velocity measurement given by GNSS.
$r_{p,k}$	position measurement noise.
$v_{p,k}$	velocity measurement noise.
$\delta_{m,k}$	relative measurement given by LO.
$r_{\delta ,k}$	relative measurement noise.
f(·)	process model.
h(·)	observation model.
${\hat{x}}_{k}$	state estimate.
$P_{k}^{xx}$	covariance matrix of state estimate.
$q_{k}$	process noise.
$Q_{k}$	covariance matrix of process noise.
$r_{k}$	output measuremnt noise.
$R_{k}$	covariance matrix of output measurement noise.
$R_{0}$	nominal covariance matrix of output measurement noise.
${\hat{R}}_{k}$	estimate of the covariance matrix of output measurement noise.
$\nu_{k}$	innovation.
$\epsilon_{k,i}^{\nu }$	normalized innovation squared.
$\xi^{\prime }$	MAV’s path.
ϑ	vector field
